# Clinical Validation of the cobas HPV Test on the cobas 6800 System for the Purpose of Cervical Screening

**DOI:** 10.1128/JCM.01239-18

**Published:** 2019-01-30

**Authors:** Marion Saville, Farhana Sultana, Michael J. Malloy, Louiza S. Velentzis, Michael Caruana, Ellen L. O. Ip, Marco H. T. Keung, Karen Canfell, Julia M. L. Brotherton, David Hawkes

**Affiliations:** a VCS Foundation, Carlton South, Victoria, Australia; b VCS Pathology, Carlton South, Victoria, Australia; c Department of Obstetrics and Gynaecology, The University of Melbourne, Parkville, Victoria, Australia; d Department of Obstetrics and Gynaecology, University of Malaya, Kuala Lumpur, Malaysia; e Melbourne School of Population and Global Health, Level 4, The University of Melbourne, Melbourne, Victoria, Australia; f Cancer Research Division, Cancer Council NSW, Woolloomooloo, NSW, Australia; g School of Public Health, Sydney Medical School University of Sydney, NSW, Australia; h Department of Pharmacology and Therapeutics, The University of Melbourne, Melbourne, Victoria, Australia; UNC School of Medicine

**Keywords:** HPV, assay, cervical, cervical intraepithelial neoplasia, cervical screening, performance

## Abstract

This study demonstrates that the clinical sensitivity, specificity, and reproducibility of the novel cobas human papillomavirus (HPV) test on the cobas 6800 system for high-risk HPV types fulfills the criteria for use in population-based cervical screening. The criteria were formulated by an international consortium, using the cobas 4800 HPV test as a validated reference assay. The cobas HPV test detected over 98% of histologically confirmed cervical intraepithelial neoplasia grade 2+ (CIN2+) lesions in women age 30 years or older, with a specificity of 98.9% compared with the reference cobas 4800 test. Both the intra- and interlaboratory agreement for the cobas HPV test were 98%. The clinical performance of the cobas HPV test is comparable to those of longitudinally validated HPV assays and fulfills the criteria for its use in primary cervical screening.

## Abstract (precis)

This study demonstrates that the clinical sensitivity, specificity, and reproducibility of the novel cobas human papillomavirus (HPV) test on the cobas 6800 system for high-risk HPV types fulfills the criteria for use in population-based cervical screening. The criteria were formulated by an international consortium, using the cobas 4800 HPV test as a validated reference assay.

## INTRODUCTION

As national cervical screening programs start to transition from cytology to human papillomavirus (HPV)-based primary testing, there is a need for high-volume clinically validated HPV assays. As of June 2018, only two countries had launched national HPV-based cervical screening programs, the Netherlands and Australia. Both countries utilize assays that test for the presence of clinically relevant levels of nucleic acids (i.e., levels correlated with the presence of an underlying high-grade cervical lesion) from the following 14 oncogenic HPV types: HPV16, HPV18, HPV31, HPV33, HPV35, HPV39, HPV45, HPV51, HPV52, HPV56, HPV58, HPV59, HPV66, and HPV68. Clinical validation has generally followed the criteria first published by an international consortium in 2009 (1). These criteria examine the assay’s clinical sensitivity and specificity for the detection of high-grade cervical disease, specifically histologically confirmed cervical intraepithelial neoplasia grade 2 or above (CIN2+) and cervical cancer. Reproducibility is also assessed with a cohort of at least 500 samples tested twice in the same laboratory (intralaboratory) and retested in an external laboratory (interlaboratory). Previous validation studies have utilized either Digene Hybrid Capture 2 or GP5+/6+-PCR-enzyme immunoassay (EIA) as their reference assay (2–4), as specified by the international consortium (1). The current validation utilized the Roche cobas 4800 HPV test as the reference assay, as its sensitivity, specificity, and reproducibility have been demonstrated to be equivalent and statistically noninferior to those of the Hybrid Capture 2 in cross-sectional comparisons (2, 5, 6). More importantly, there have been a number of large high-quality prospective studies which have demonstrated that the cobas 4800 HPV test has acceptable clinical performance in longitudinal clinical trials (7–9) against cytological and histological measures of cervical disease rather than just HPV results. The cobas 4800 HPV test has also demonstrated 100% proficiency in detecting the targeted 14 HPV types in both the 2013 and 2014 HPV LabNet International proficiency studies (10).

The Roche cobas HPV test, which can be run on either the Roche cobas 6800 or 8800 system, has yet to be clinically validated for sensitivity, specificity, or reproducibility. This assay tests for the same 14 HPV types as the cobas 4800 HPV, Abbott RealTi*m*e high-risk HPV, BD Onclarity HPV, Seegene Anyplex II HR HPV, Cepheid Xpert HPV, Hologic Aptima HPV, Greiner PapilloCheck HPV-screening, and Self-Screen HPV-Risk assays. All Roche cobas HPV assays (the established 4800 and the novel HPV test run on 6800/8800) also give partial genotyping by separately identifying HPV16 and HPV18, with the results for the remaining 12 types reported in a combined “pool.” The novel cobas HPV assays also utilize a human beta-globin gene as a control for inhibition and cellularity. The cobas HPV test for use on the 6800 and 8800 systems is currently being validated for HPV primary screening in a U.S.-based clinical trial.

## MATERIALS AND METHODS

### Study population.

The study population was drawn from women who participated in the Compass Main Trial, a population-based cervical screening trial comparing primary HPV testing with cytology-based cervical screening in Australia in women age 25 to 64 years (11). Women in the Compass Trial had their cervical screening sample collected into liquid-based cytology medium (PreservCyt) and consented to having their samples biobanked following completion of all testing that was specified in the trial protocol.

From this pool of samples, we selected samples from women age at least 30 years for our analyses following the principles published by Meijer and colleagues (1) and at a power of 80%, as has been used in previous clinical validations (2, 4).

**(i) Clinical sensitivity.** For clinical sensitivity, we identified a set of 60 cervical screening samples from women who subsequently had histologically confirmed cervical intraepithelial neoplasia grade 2 (CIN2), adenocarcinoma *in situ* (AIS), or worse within 6 months of subsequent follow-up (referred to here as “cases”). The median age of cases was 33 years (range, 30 to 57 years). During the period of this study, the Compass Trial was only recruiting women born after 30 June 1980, which, when combined with the minimum inclusion age of 30, meant that nearly all participants were between 30 and 35 years of age. Only samples with a valid test result on the cobas 4800 assay were included in the cases. In the Compass Trial, specimens returned invalid results on the cobas 4800 HPV assay at a rate of <0.2%. The cases comprised 21 CIN2, 3 CIN2/CIN3, 22 CIN3, 10 CIN3/carcinoma *in situ* (CIS), 3 AIS, and 1 mixed AIS/CIS.

**(ii) Clinical specificity.** For clinical specificity, we identified a set of 805 cervical screening samples from women who had no evidence of histologically confirmed CIN2, AIS, or worse within 6 months of subsequent follow-up (referred to as controls). We excluded samples from any woman whose test was undertaken in follow-up of a previous abnormality, a positive HPV test, or as “test-of-cure” in the follow-up of histologically confirmed and treated high-grade squamous intraepithelial lesion (HSIL). The median age of controls was 33 years (range, 30 to 69 years). Only samples with a valid test result on the cobas 4800 HPV test were included in the controls.

**(iii) Intralaboratory reproducibility.** For intralaboratory reproducibility, we identified a set of 500 cervical samples, of which 150 samples were positive on the cobas 4800 HPV test. All 500 samples were tested twice at Victorian Cytology Service (VCS) Pathology using the novel cobas HPV test, with a median interval of 150 days (range, 17 to 333 days).

**(iv) Interlaboratory reproducibility.** For interlaboratory reproducibility, the samples were then tested at another laboratory (National Serology Reference Laboratory [NRL], Victoria, Australia) using the novel cobas HPV test. Only samples with a valid test result on both the cobas 6800 and cobas 4800 instruments were included in the analysis. The median interval between testing at VCS Pathology and NRL was 60 days (range, 32 to 66 days).

### cobas 4800 HPV test.

The cobas 4800 HPV test is a real-time PCR assay that detects the presence of 14 HPV types, with HPV16 and HPV18 detected individually and a pool of 12 other HPV types (HPV31, HPV33, HPV35, HPV39, HPV45, HPV51, HPV52, HPV56, HPV58, HPV59, HPV66, and HPV68). We used the Roche cobas 4800 HPV test as the reference assay, as its sensitivity, specificity, and reproducibility have been demonstrated to be equivalent and statistically noninferior to Hybrid Capture 2 in cross-sectional comparisons (2, 5). In addition, there have been a number of publications which have demonstrated that Roche cobas 4800 HPV test has acceptable clinical performance in longitudinal clinical trials (7–9).

### cobas HPV test.

The novel cobas HPV test is similar to the cobas 4800 HPV test in that it uses real-time PCR to detect the presence of the same 14 HPV types with the same partial genotyping. The main difference is that the assay is of a shorter duration by over an hour (final run time, 3.5 h), which indicates that changes have been made to the chemistry of the assay. The cobas HPV test for use on the 6800 and 8800 systems is currently being validated for HPV primary screening in a U.S.-based clinical trial.

### Statistical analysis.

The results using the novel cobas HPV test on the cobas 6800 system were compared to the outcomes found by the cobas 4800 HPV test. We estimated the clinical sensitivity and specificity (including 95% confidence intervals) of the novel cobas HPV and cobas 4800 HPV tests for detection of CIN2+ lesions separately. We also examined type-specific HPV detection using the two assays in cases and controls (stratified by result, HPV16/18 positive or other HPV positive). We then compared the clinical sensitivity and specificity values of the novel cobas HPV test with those of the cobas 4800 HPV test using the noninferiority score test. The relative sensitivity threshold for CIN2+ is defined to be at least 90% of the reference validated assay, and the relative specificity threshold for CIN2+ is at least 98% as proposed in the consensus guidelines (1). For intra- and interlaboratory analyses, we calculated the overall agreement with 95% binomial exact confidence intervals and determined the intralaboratory reproducibility and interlaboratory agreement using Gwet’s (AC_1_) agreement coefficient (16). For interlaboratory agreements, we compared the first and second results for the reproducibility cohort from VCS Pathology with the NRL results separately. A lower confidence bound for agreement of not less than 87% and an AC_1_ score of at least 0.5 was used as a threshold. The level of significance (α) was set at 0.05. Microsoft Excel 2013 was used for calculations of noninferiority scores using the formulae specified in the guidelines paper (1). All other statistical analyses were performed using STATA version 12.1 (Stata Corp., College Station, TX, USA).

This study was approved by the Bellberry Human Research Ethics Committee.

## RESULTS

### Clinical sensitivity and specificity.

Of the 60 cases, 59 were positive for HPV on the novel cobas HPV test, resulting in a clinical sensitivity of 98.3% (95% confidence interval [CI], 91.1% to 99.9%). In comparison, all 60 cases were positive on the cobas 4800 HPV test, giving a clinical sensitivity of 100% (95% CI, 93.9% to 100%; Table 1). One sample, a CIN2 lesion, which tested negative on the novel cobas HPV test was positive for non-HPV16/18 HPV on the cobas 4800 HPV test (Table 2). Of the 805 controls, 712 were negative for HPV on the novel cobas HPV test, resulting in a clinical specificity of 88.4% (95% CI, 86% to 90.6%; Table 1).

**TABLE 1:** HPV test findings among 865 cervical samples collected in the Compass clinical trial, stratified by the presence or absence of histologically confirmed CIN2+ lesions

Clinical status	cobas HPV test result	cobas 4800 HPV test result		Total
		Negative	Positive	
Controls (<CIN2)	Negative	712	0	712
	Positive	8	85	93
	Total	720	85	805
Cases (≥CIN2)	Negative	0	1	1
	Positive	0	59	59
	Total	0	60	60

**TABLE 2:** Type-specific HPV detection using the cobas HPV test in relation to the cobas 4800 HPV test stratified by the presence or absence of histologically confirmed CIN2+ lesion

Clinical status	cobas HPV test result	cobas 4800 HPV test result (no.)							Total no.
		HPV negative	HPV16	HPV18	Other HPV	HPV16 and other HPV	HPV18 and other HPV	HPV16 and HPV18	
Controls (<CIN2)	HPV negative	712	0	0	0	0			712
	HPV16	5	2	0	0	0			7
	HPV18	2	0	3	0	0			5
	Other HPV	1	0	0	78	0			79
	HPV16 and other HPV	0	0	0	0	2			2
	Total	720	2	3	78	2			805
Cases (CIN2+)	HPV negative	0	0	0	1	0	0	0	1
	HPV16	0	14	0	0	0	0	0	14
	HPV18	0	0	5	0	0	0	0	5
	Other HPV	0	0	0	31	0	0	0	31
	HPV16 and other HPV	0	0	0	0	7	0	0	7
	HPV18 and other HPV	0	0	0	0	0	1	0	1
	HPV16 and HPV18	0	0	0	0	0	0	1	1
	Total	0	14	5	32	7	1	1	60

The clinical specificity of cobas 4800 HPV test for CIN2+ was 89.4% (95% CI, 87.1% to 91.5%) (720/805 controls). Both clinical sensitivity and specificity of the novel cobas HPV test were noninferior to the cobas 4800 HPV test (*P* = 0.016 and *P* = 0.04). Relative to the cobas 4800 HPV test, the novel cobas HPV test had a specificity of 98.9% for histologically confirmed CIN2+ lesions in women age 30 years or older. Among the controls, 93 (11.6%) were positive on the cobas 6800 system, and 85 (10.6%) were positive on cobas 4800 test. Of the 8 controls that tested positive on the cobas 6800 system but were negative on the cobas 4800 test, 5 were HPV16, 2 were HPV18, and 1 was non-HPV16/18 positive (Table 2). All eight samples remained negative on retesting with the cobas 4800 test.

### Intralaboratory reproducibility and interlaboratory agreement.

The intralaboratory reproducibility over time was 98.2% (491/500; 95% CI, 96.6% to 99.2%), with an AC_1_ value of 0.97 (Table 3). The interlaboratory agreement was 98.4% (492/500; 95% CI, 96.9% to 99.3%), with an AC_1_ value of 0.97 for the first test and agreement of 99% (495/500; 95% CI, 97.7% to 99.7%) and an AC_1_ value of 0.98 for the second test (Table 4). Both the intralaboratory reproducibility over time and the interlaboratory agreement fulfilled the validation guidelines of a lower confidence bound of percentage of agreement that was higher than 87% and a corresponding AC_1_ value that was higher than 0.5 (see Tables SA to SF in the supplemental material).

**TABLE 3:** Intralaboratory reproducibility over time of cobas HPV test for HPV detection

VCS, first test	VCS, second test result (no.)		
	HPV negative	HPV positive	Total
HPV negative	344	6	350
HPV positive	3	147	150
Total	347	153	500

**TABLE 4:** Interlaboratory agreement of cobas HPV test for HPV detection

VCS test result	NRL result (no.)		
	HPV negative	HPV positive	Total
First test			
HPV negative	344	6	350
HPV positive	2	148	150
Total	346	154	500
Second test			
HPV negative	344	3	347
HPV positive	2	151	153
Total	346	154	500

### Discordant results.

Three samples that tested positive in the first test and negative for their repeat test were all positive for non-HPV16/18 HPV, with two of the three testing negative in the NRL (the other one was positive for non-HPV16/18 HPV in the NRL). Of the six samples that tested positive in the repeated test but were negative on the original test, 3 were HPV16, 1 was HPV18, and 2 were non-HPV16/18 HPV. The three HPV16-positive samples and the one HPV18-positive sample that were detected in the second test were tested by the novel cobas HPV test at the NRL (see Tables SA to SF).

## DISCUSSION

In this study, the clinical performance of the novel cobas HPV test processed on the cobas 6800 system was compared to the validated cobas 4800 HPV test in a cohort of screening participants age 30 years or older. The clinical sensitivity and specificity for histologically confirmed CIN2+ of the novel cobas HPV test were noninferior to those for the cobas 4800 HPV test, which we consider a validated reference assay. The novel cobas HPV test also demonstrated high levels of intra- and interlaboratory reproducibility. The use of the cobas 4800 HPV test as the reference assay also allowed the comparison of partially genotyped results. The benefit of being able to undertake this comparison is relevant for some national cervical screening programs, like Australia’s, which use partial genotyping as part of the clinical management of HPV-positive women.

The strengths of this study are the use of a representative screening population recruited from routine practice in Australia, the large number of samples of known clinical history provided by the Victorian Cytology Service Registry, which records primary and follow-up test results, and the use of agreed published international methodological criteria for assessment. The main limitation is that we were unable to utilize either of the previous reference assays (Digene Hybrid Capture 2 or GP5+/6+-PCR-EIA) as our comparator, as neither are in current use in Australia. The current study represents the first validation utilizing the framework of the Meijer criteria protocol using a reference assay other than the Hybrid Capture 2 or GP5+/6+ EIA, which were described in the original manuscript (1).

According to requirements for laboratories reporting tests for the National Cervical Screening Program from Australia’s National Pathology Accreditation Advisory Council (12), HPV screening assays in use in Australia need to include a control, which allows samples with insufficient sample (cells) to be identified and not be given a false-negative result based on the absence of HPV alone, and the HC2 assay does not meet this requirement. We selected the cobas 4800 HPV test as our comparator due to its documented performance comparable to that of HC2, its availability in multiple laboratories in Australia which facilitates interlaboratory assessment, and because we consider that its performance is now sufficiently prospectively validated in published data. In the ATHENA study, the sensitivity and specificity of the cobas 4800 HPV test in detecting CIN2+ and CIN3+ in women with atypical squamous cells of undetermined significance (ASC-US) were comparable to findings using HC2 in the same population (13). The results at the end of the study also showed that the cumulative incidence rate of CIN3+ for HPV-negative women as tested by the cobas 4800 HPV test was 0.3% (95% CI, 0.1 to 0.7) compared to 0.8% (95% CI, 0.5 to 1.1) after a negative cytology test and 0.3% (95% CI, 0.1 to 0.6) following negative results from cytology and HPV cotesting (14).

Although there is a body of evidence that the cobas 4800 system is equivalent in sensitivity and specificity to the Hybrid Capture 2, as demonstrated using the Meijer criteria protocol, several other assays can make the same claim. If a different clinically validated assay, such as the Abbott RealTi*m*e or Hologic Aptima, were to be used as the comparator, there would likely be variation in sensitivity and specificity. We support the use of the cobas 4800 as a reference assay for clinical validation based on its performance in detecting CIN2+ while maintaining high levels of specificity. A recent manuscript (15) used the cobas 4800 HPV assay as the comparator for demonstrating the longitudinal value of the Hologic Aptima in detecting HPV infections that would lead to CIN3+ up to 7 years later. Differences between the two assays were not statistically significant, but the cobas 4800 assay was both more sensitive and had a higher negative predictive value, thus demonstrating its value as a clinical benchmark.

We consider that the novel cobas HPV test processed on the cobas 6800 system will be a useful addition to the options available for primary HPV screening in Australia and elsewhere given its automated sample preparation and high-throughput (over 1,000 samples in 24 h), although the size and weight (∼1,700 kg) of the cobas 6800 system make it more difficult to fit into a laboratory compared to other smaller-volume systems. Prospective clinical validation of its performance in real-world screening will follow in the short term, as comprehensive and complete data capture of screening results and subsequent diagnoses occur as part of Australia’s renewed cervical screening program. In particular, confirmation of the adequacy of the assay’s specificity, when combined with reflex cytology in routine use, will be important. In conclusion, the novel cobas HPV test processed on the cobas 6800 system fulfills all requirements, as outlined by an international consortium (1), for being a clinically validated assay that can be used in primary cervical cancer screening.

## Supplementary Material

Supplemental file 1
