# Supplementary material for: Clinical Validation of the cobas HPV Test on the cobas 6800 System for the Purpose of Cervical Screening
Source: J Clin Microbiol. 2019 Jan 30;57(2):e01239-18. doi: 10.1128/JCM.01239-18 (PMC6355513; doi:10.1128/JCM.01239-18)
Supplement: Supplemental file 1 [file 71b33d950087edcd7477a0ac3289a619_JCM.01239-18-s0001.pdf]

Supplementary Tables: Appendix

Table A: Intra-laboratory reproducibility of type specific HPV over time using cobas HPV test

VCS, First Test	VCS, Second Test							
	HPV Negative	HPV16	HPV18	Other HPV	HPV16 & HPV18	HPV16 & other HPV	HPV18 & other HPV	Total
HPV negative	344	3	1	2	0	0	0	350
HPV positive								
HPV 16	0	18	0	0	0	0	0	18
HPV 18	0	0	8	0	0	0	0	8
HPV Other	3	0	0	110	0	0	0	113
HPV 16 & HPV 18	0	0	0	0	2	0	0	2
HPV 16 & other HPV	0	0	0	1	0	7	0	8
HPV 18 & other HPV	0	0	0	0	0	0	1	1
**Total**	**347**	**21**	**9**	**113**	**2**	**7**	**1**	**500**

Overall genotype agreement= 98% (490/500; 95% CI: 96.4 to 99.0) and AC_1 value = 0.98

Table B: Clinical agreement in HPV detection (intra-laboratory)

		cobas HPV test First Test			
		HPV16/18 (± Other HPV types)	Other HPV types (non-HPV16/18)	HPV negative	Total
cobas HPV test Second Test	HPV16/18 (± Other HPV types)	36	0	4	40
	Other HPV types (non-HPV16/18)	1	110	2	113
	HPV negative	0	3	344	347
	**Total**	**37**	**113**	**350**	**500**

Overall agreement = 98% (490/500; 95% CI: 96.4% to 99.0%) and AC_1 value = 0.97

Table C: Inter-laboratory agreement of type-specific HPV using cobas HPV test

VCS, First Test	NRL							Total
	HPV Negative	HPV 16	HPV 18	HPV Other	HPV 16 & HPV 18	HPV 16 & other HPV	HPV 18 & other HPV	
HPV negative	344	4	1	1	0	0	0	350
HPV positive								
HPV 16	0	18	0	0	0	0	0	18
HPV 18	0	0	8	0	0	0	0	8
HPV Other	2	0	0	110	0	0	1	113
HPV 16 & HPV 18	0	0	0	0	2	0	0	2
HPV 16 & other HPV	0	0	0	0	0	8	0	8
HPV 18 & other HPV	0	0	0	0	0	0	1	1
**Total**	**346**	**22**	**9**	**111**	**2**	**8**	**2**	**500**

Overall genotype agreement= 98.2% (491/500; 95% CI: 96.6 to 99.2) and AC_1 value = 0.98

Table D: Inter-laboratory agreement of type-specific HPV using cobas HPV test

VCS, Second Test	NRL							Total
	HPV Negative	HPV 16	HPV 18	HPV Other	HPV 16 & HPV 18	HPV 16 & other HPV	HPV 18 & other HPV	
HPV negative	344	1	0	2	0	0	0	347
HPV positive								
HPV 16	0	21	0	0	0	0	0	21
HPV 18	0	0	9	0	0	0	0	9
HPV Other	2	0	0	109	0	1	1	113
HPV 16 & HPV 18	0	0	0	0	2	0	0	2
HPV 16 & other HPV	0	0	0	0	0	7	0	7
HPV 18 & other HPV	0	0	0	0	0	0	1	1
**Total**	**346**	**22**	**9**	**111**	**2**	**8**	**2**	**500**

Overall genotype agreement= 98.6% (493/500; 95% CI: 97.1 to 99.4) and AC_1 value = 0.98

Table E: Clinical agreement in HPV detection (inter-laboratory)

		Roche cobas HPV test VCS Pathology First Test			
		HPV 16 and/or 18 (± other HPV types)	Other HPV types (not HPV 16/18)	HPV negative	Total
Roche cobas HPV test NRL	HPV 16 and/or 18 (± other HPV types)	37	1	5	43
	Other HPV types (not HPV 16/18)	0	110	1	111
	HPV negative	0	2	344	346
	Total	37	113	350	500

Overall agreement= 98.2% (491/500; 95% CI: 96.6% to 99.2%) and AC_1 value = 0.98

Table F: Clinical agreement in HPV detection (inter-laboratory)

		Roche cobas HPV test VCS Pathology Second Test			
		HPV 16 and/or 18 (± other HPV types)	Other HPV types (not HPV 16/18)	HPV negative	Total
Roche cobas HPV test NRL	HPV 16 and/or 18 (± other HPV types)	40	2	1	43
	Other HPV types (not HPV 16/18)	0	109	2	111
	HPV negative	0	2	344	346
	Total	40	113	347	500

Overall agreement= 98.6% (493/500; 95% CI: 97.1% to 99.4%) and AC_1 value = 0.98
